# Supplementary material for: Young infants display heterogeneous serological responses and extensive but reversible transcriptional changes following initial immunizations
Source: Nat Commun. 2023 Dec 2;14:7976. doi: 10.1038/s41467-023-43758-2 (PMC10693608; doi:10.1038/s41467-023-43758-2)
Supplement: Supplementary file 1 — Supplementary Information [file 41467_2023_43758_MOESM1_ESM.pdf]

Young infants display heterogeneous serological responses and extensive but reversible transcriptional changes following initial immunizations

Nima Nouri[1,2,#], Raquel Giacomelli Cao[3,4,#], Eleonora Bunsow[3,#], Djamel Nehar-Belaid[1,#], Radu Marches[1], Zhaohui Xu[3,5], Bennett Smith[3], Santtu Heinonen[3,6], Sara Mertz[3], Amy Leber[7], Gaby Smits[8], Fiona van der Klis[8], Asunción Mejías[3,4,5], Jacques Banchereau[1,9,†], Virginia Pascual[10,†,*], Octavio Ramilo[3,4,5,†,*]

[#] These authors contributed equally

[†] These authors jointly supervised the work

* Correspondence: Octavio Ramilo (octavio.ramilo@stjude.org) and Virginia Pascual (vip2021@med.cornell.edu)

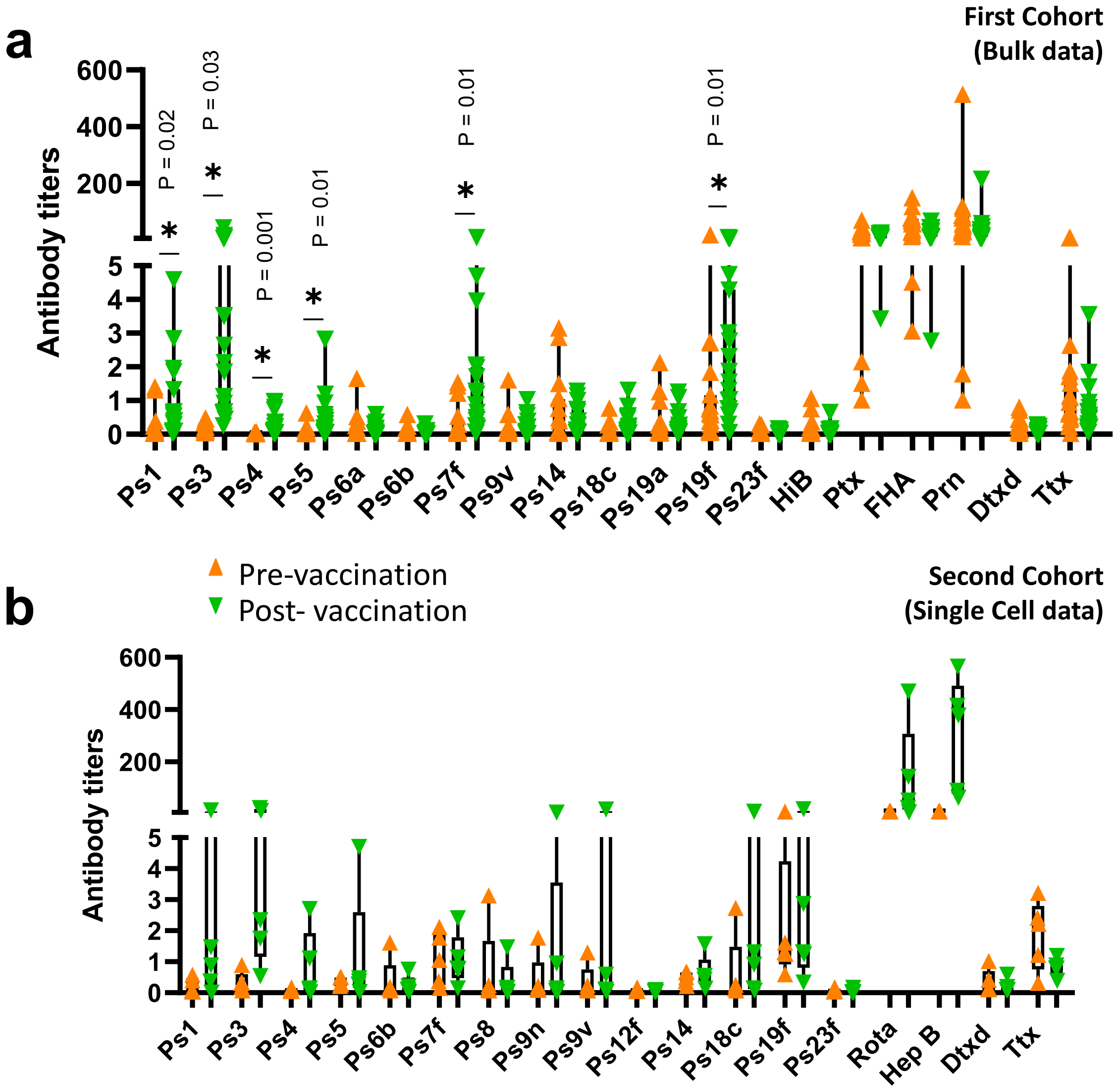

Supplementary Figure 1: Antibody response in 2-month-old infants from bulk transcriptome cohort (A) and single sell cohort (B). Antibody titers (y-axis) before vaccination (day 0; orange triangles) and approximately 4 weeks later (green inverted triangles) against different vaccine antigens (x-axis) in each individual infant (n=19 with serologic responses from the first cohort and n=5 from the second cohort). An asterisk (*) indicates a significant difference of unadjusted p<0.05 (labels), calculated by a two-sided t-test. Ps: Pneumococcus, Rota: Rotavirus, Hep B: Hepatitis B, Dtxd: Diphtheria toxoid, HiB: Haemophilus influenza type b, Ptx: Pertussis toxin, FHA: Filamentous hemagglutinin, Prn: Pertactin. Please note that the antibody titers measured were slightly different in the two separate cohorts.

a

Sample	Age	Gender	Race/Ethnicity	Infant	Time point
S1	2.17	F	W	I1	A
S2	2.40	F	W	I1	B
S3	3.23	F	W	I1	C
S4	1.87	F	W	I2	A
S5	2.17	F	W	I2	B
S6	2.97	F	W	I2	C
S7	1.93	M	Biracial	I3	A
S8	2.37	M	Biracial	I3	B
S9	3.03	M	Biracial	I3	C
S10	1.93	F	W	I4	A
S11	2.17	F	W	I4	B
S12	2.83	F	W	I4	C
S13	1.83	F	AA	I5	A
S14	2.07	F	AA	I5	B
S15	2.90	F	AA	I5	C
S16	2.13	F	AA	I6	A
S17	2.40	F	AA	I6	B
S18	3.33	F	AA	I6	C

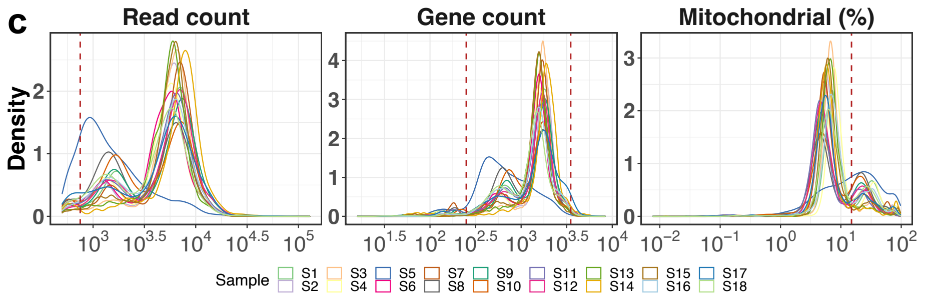

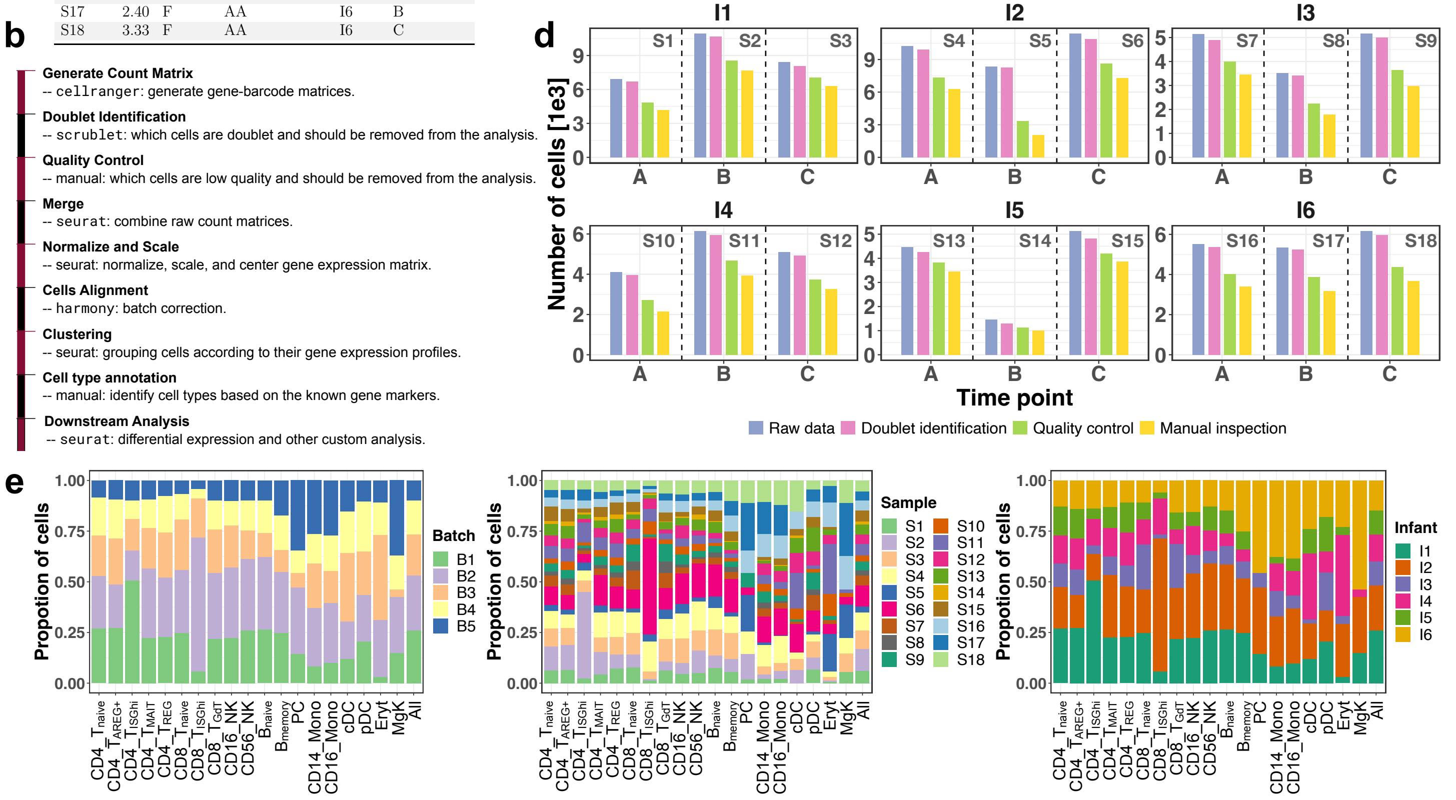

Supplementary Figure 2: Overview of single-cell gene expression analysis. (a) Dataset demographics: F: Female, M: Male, W: White, AA: African American. Ages are shown in months. **(b)** Overview of the computational pipeline. **(c)** Quality control metrics: number of reads per cell (left panel), number of expressed genes per cell (middle panel), and mitochondrial percentage (right panel) for each of the n=18 color-coded samples. Dashed lines indicate the thresholds used in quality control (Read count = 750; Genes count between 250 and 3500; and percentage of mitochondrial < 15%). **(d)** Number of cells before and after filtration, including doublet removal, quality control removal, and manual removal. **(e)** Proportion of cells per batch (left panel), per sample (middle panel), and in each individual infant (right panel) for each of the different cell types.

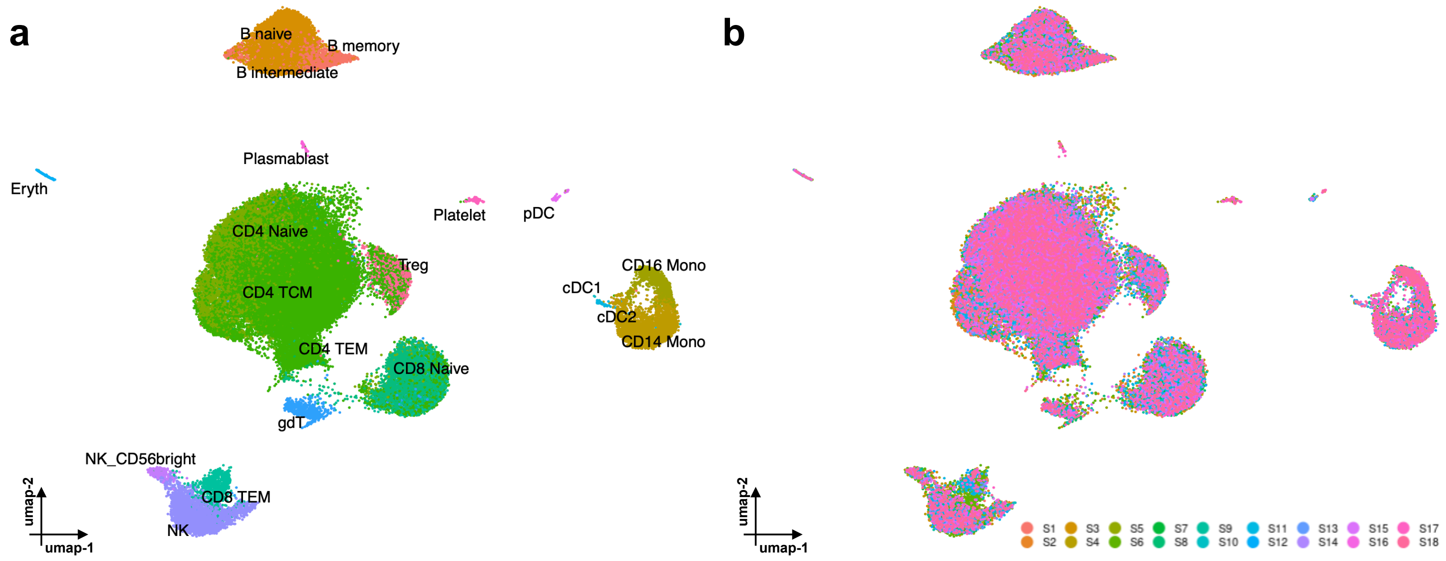

Supplementary Figure 3: **Additional supporting UMAPs**. **(a)** Automated reference-based (Stuart 2019) annotation results using Azimuth web application (Hao 2021). **(b)** UMAPs demonstrating good overlap between different samples (color-coded cells grouped by sample).

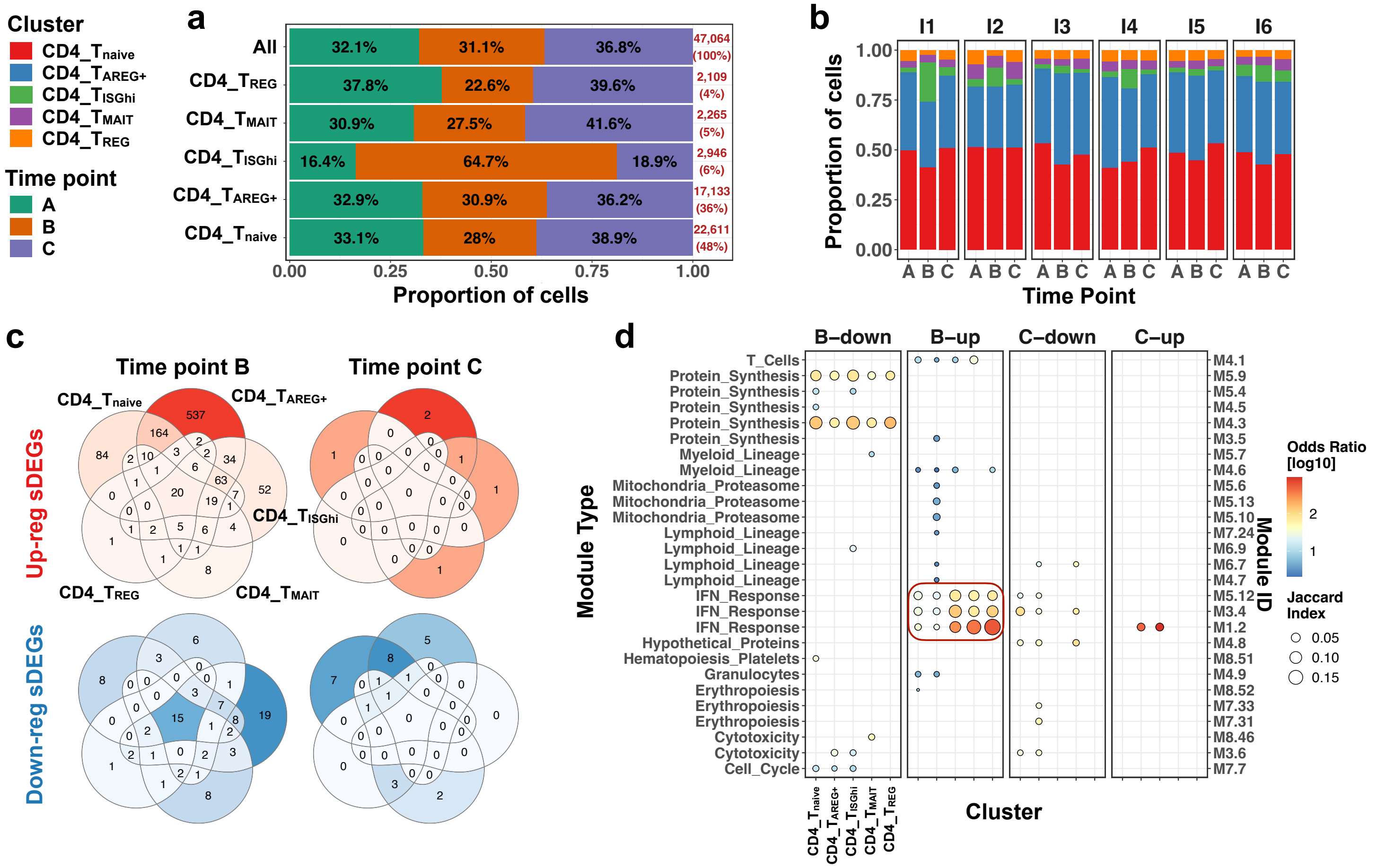

Supplementary Figure 4: CD4+ T cells supplementary panel. Subcluster (SC) and time-point color codes are shown in the top-left. **(a)** Proportion of cells per SC at each time point. **(b)** Proportion of cells at each time point for each SC and infant. **(c)** Venn-diagram showing the number of significantly differentially expressed genes (sDEGs) for each comparison among SCs per time point and regulation type (up/down-regulated). sDEGs are determined as those with a log2-fold-change>0.25 and adjusted p<0.05, calculated using two-sided Wilcoxon rank-sum tests. **(d)** Significant overlaps (determined by Fisher's exact test, p<0.05) between sDEGs and modules for each time point and regulation type within each SC. The odds ratio (color-bar legend) represents the strength of association, and the Jaccard index (legend circles) measures the similarity between two lists.

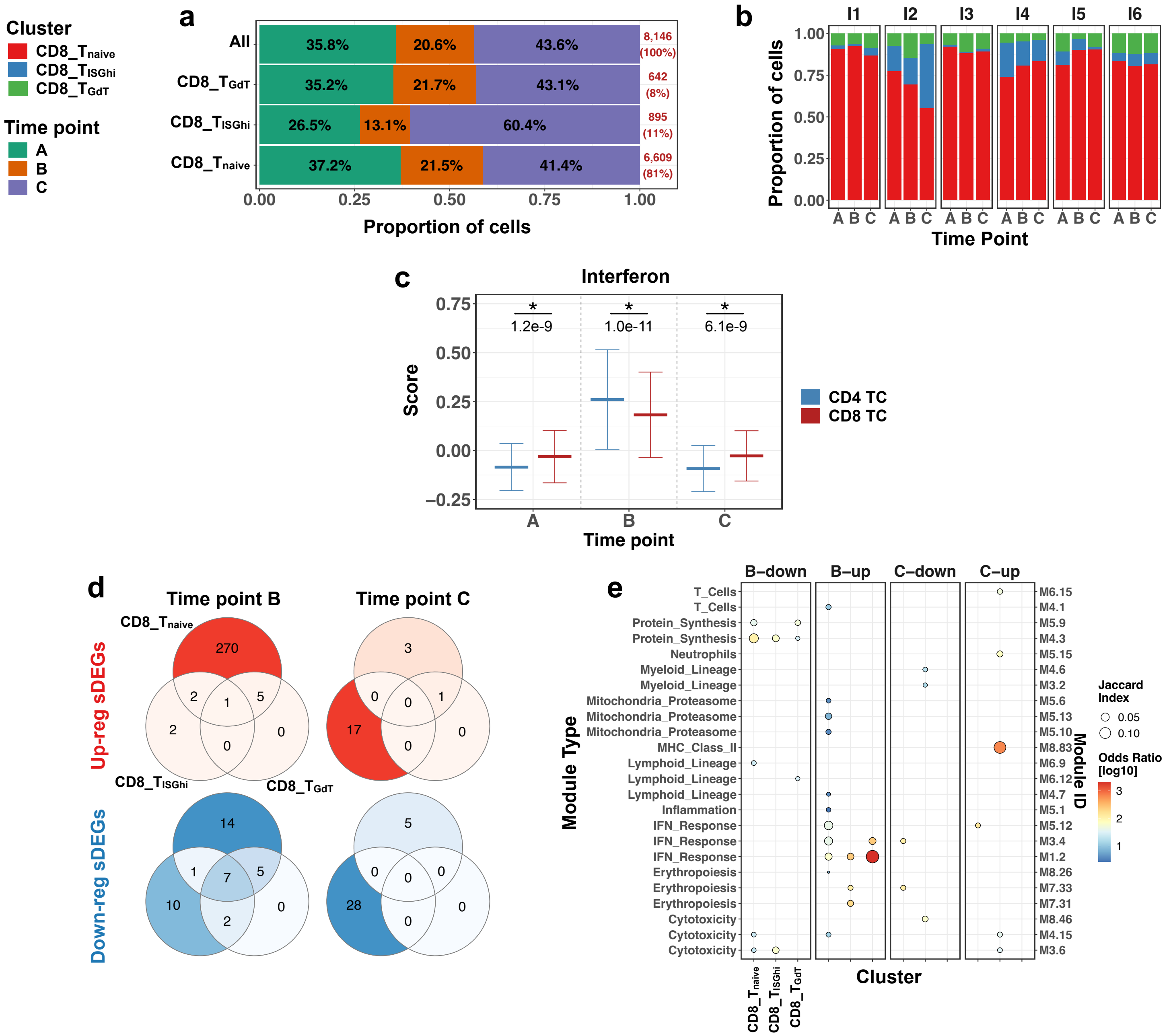

Supplementary Figure 5: CD8+ T cells supplementary panel. Subcluster (SC) and time-point color codes are shown in the top-left. **(a)** Proportion of cells per SC at each time point. **(b)** Proportion of cells at each time point for each SC and infant. **(c)** Interferon score comparison between color-coded CD4+ and CD8+ TCs per time point. Standard deviations indicate variation in scores across cells. Horizontal bars indicate the mean. Asterisk (*) indicates a significant difference of unadjusted $p<0.05$ calculated by a two-sided t-test. **(d)** Venn-diagram showing the number of significantly differentially expressed genes (sDEGs) for each comparison among SCs per time point and regulation type (up/down-regulated). sDEGs are determined as those with a log2-fold-change>0.25 and adjusted $p<0.05$, calculated using two-sided Wilcoxon rank-sum tests. **(e)** Significant overlaps (determined by Fisher's exact test, $p<0.05$) between sDEGs and modules for each time point and regulation type within each SC. The odds ratio (color-bar legend) represents the strength of association, and the Jaccard index (legend circles) measures the similarity between two lists.

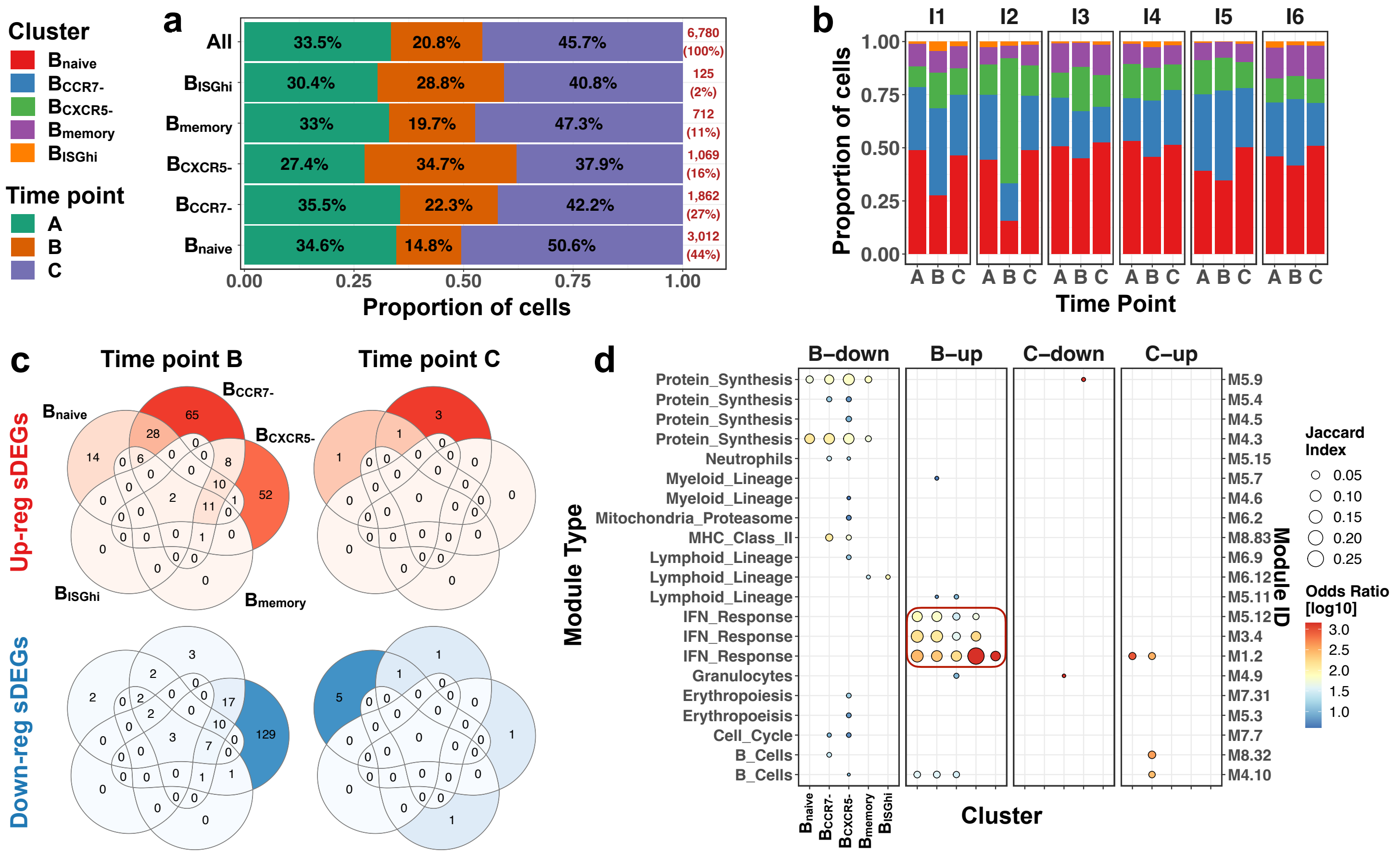

Supplementary Figure 6: B cells supplementary panel. Subcluster (SC) and time-point color codes are shown in the top-left. **(a)** Proportion of cells per SC at each time point. **(b)** Proportion of cells at each time point for each SC and infant. **(c)** Venn-diagram showing the number of significantly differentially expressed genes (sDEGs) for each comparison among SCs per time point and regulation type (up/down-regulated). sDEGs are determined as those with a log2-fold-change>0.25 and adjusted p<0.05, calculated using two-sided Wilcoxon rank-sum tests. **(d)** Significant overlaps (determined by Fisher's exact test, p<0.05) between sDEGs and modules for each time point and regulation type within each SC. The odds ratio (color-bar legend) represents the strength of association, and the Jaccard index (legend circles) measures the similarity between two lists.

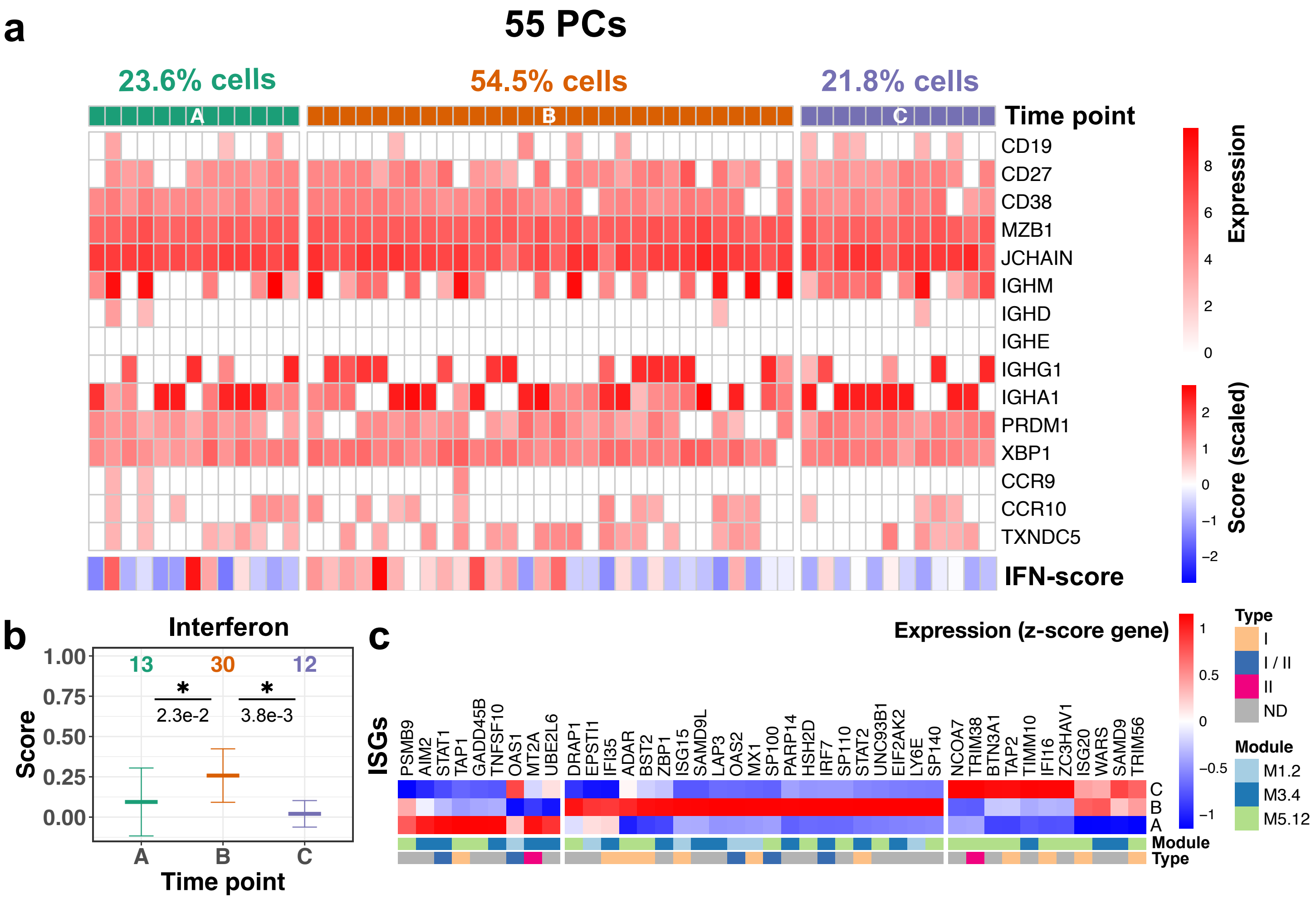

Supplementary Figure 7: Vaccinated infants display an expansion of plasma cells expressing interferon-stimulated genes at day 7 post-vaccination. **(a)** Expression levels of selected genes for each cell. Scaled interferon scores calculated for each cell are shown in the bottom row. Proportion of cells for each time point is indicated by a color-coded label on top. **(b)** Interferon scores calculated for each cell per time point. Error bars indicate standard deviation across cells, and the horizontal bar represents the mean. An asterisk (*) indicates a significant difference between two consecutive time points, determined using two-sided t-tests, with unadjusted p<0.05 (labels). The number of cells for each time point is shown by a color-coded label on top. **(c)** Average expression levels of the top 30 Interferon-stimulated genes (ISGs) per each time point. The color keys at the bottom represent gene associations.

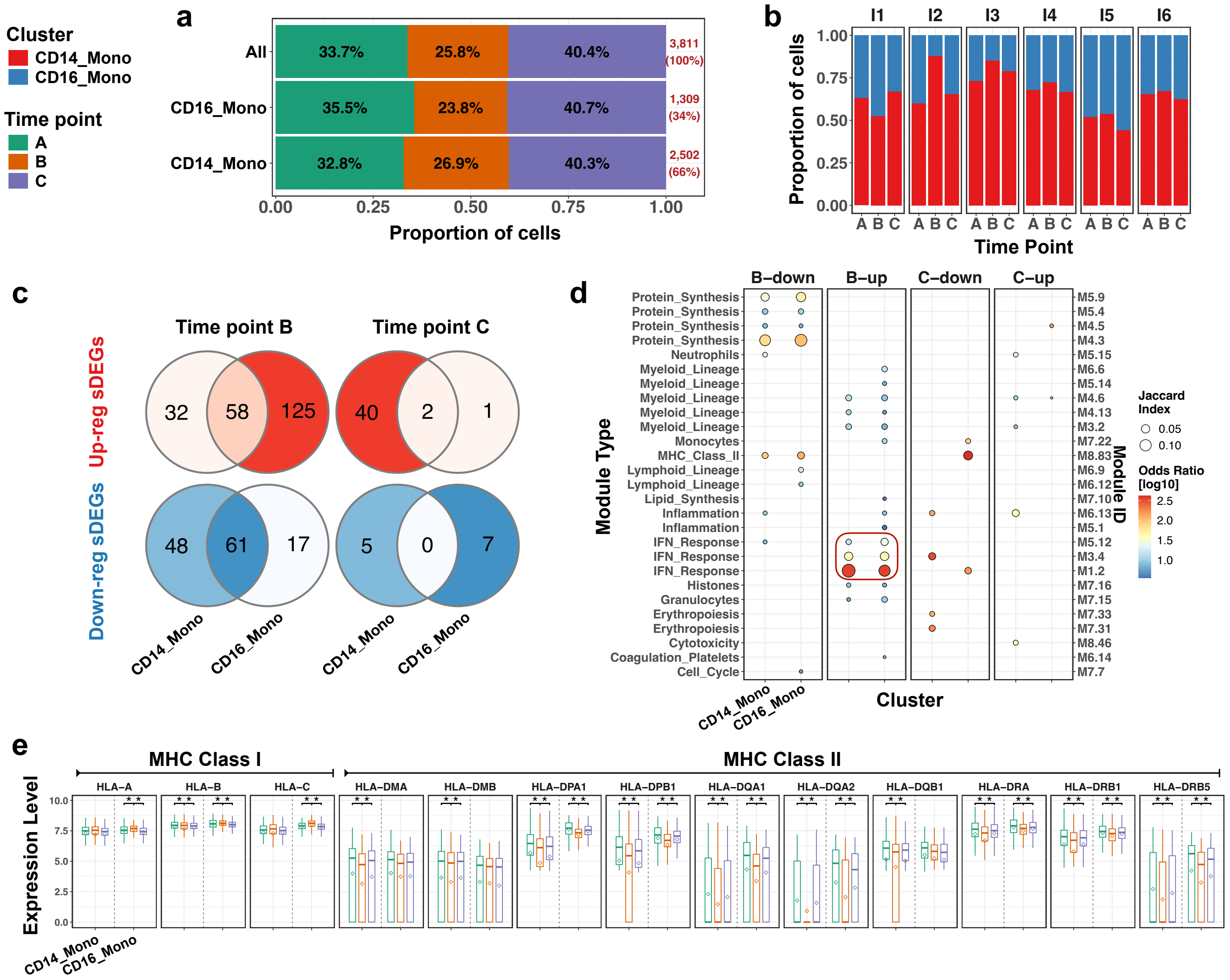

Supplementary Figure 8: Monocytes supplementary panel. Subcluster (SC) and time-point color codes are shown in the top-left. **(a)** Proportion of cells per SC at each time point. **(b)** Proportion of cells at each time point for each SC and infant. **(c)** Venn-diagram showing the number of significantly differentially expressed genes (sDEGs) for each comparison among SCs per time point and regulation type (up/down-regulated). sDEGs are determined as those with a log2-fold-change>0.25 and adjusted p<0.05, calculated using two-sided Wilcoxon rank-sum tests. **(d)** Significant overlaps (determined by Fisher's exact test, p<0.05) between sDEGs and modules for each time point and regulation type within each SC. The odds ratio (color-bar legend) represents the strength of association, and the Jaccard index (legend circles) measures the similarity between two lists. **(e)** MHC class I and II related genes expression pattern over time for each SC. The results are depicted in boxplots for all cells, in which the upper and lower bounds represent the 75% and 25% percentiles, respectively. The center bars indicate the medians, and the whiskers denote values up to 1.5 interquartile ranges above the 75% or below the 25% percentiles. The diamonds indicate the mean. An asterisk (*) indicates a significant difference between two consecutive time points, determined using two-sided t-tests, with unadjusted p<0.05.

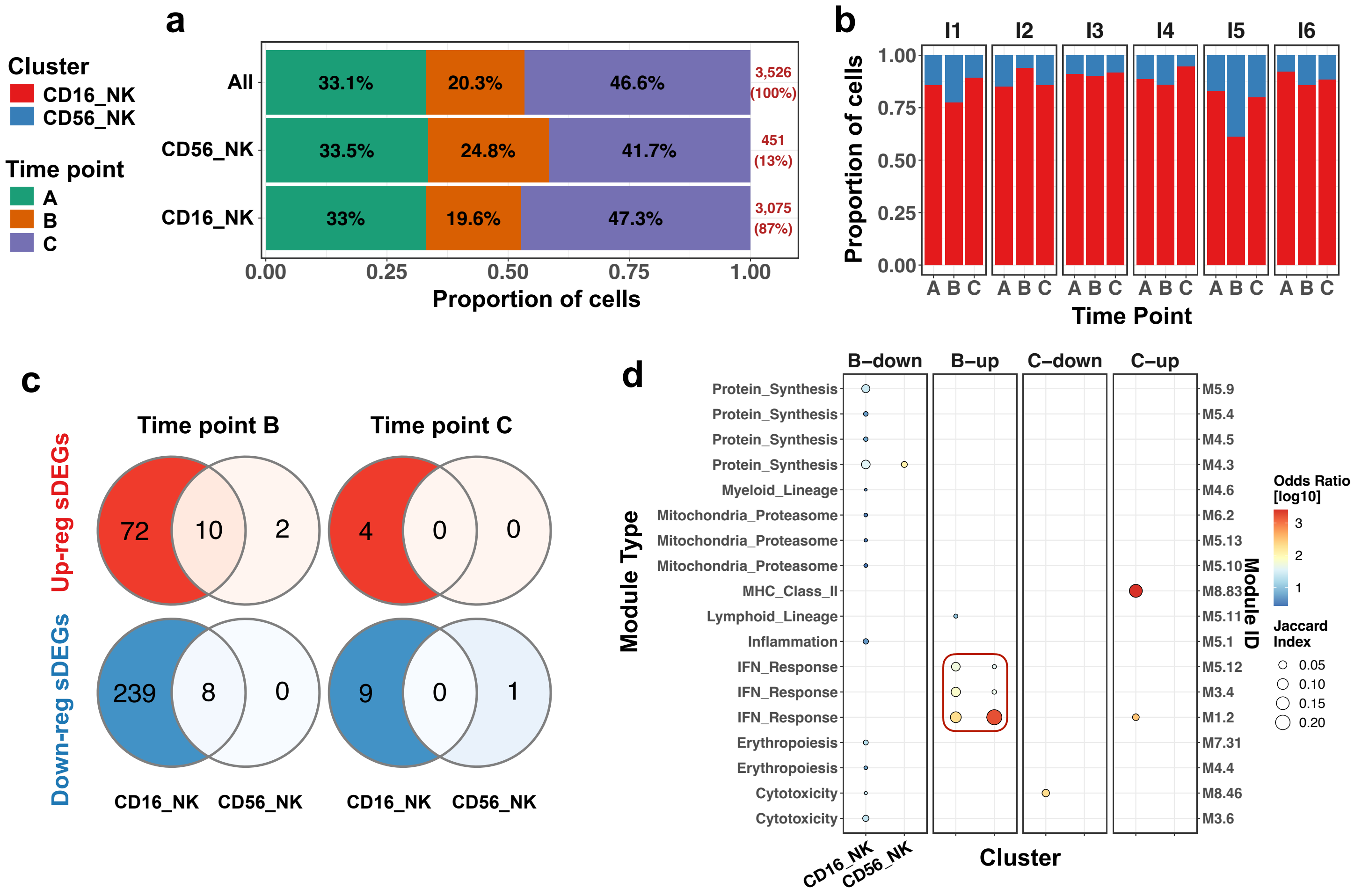

Supplementary Figure 9: Natural killer cells supplementary panel. Subcluster (SC) and time-point color codes are shown in the top-left. **(a)** Proportion of cells per SC at each time point. **(b)** Proportion of cells at each time point for each SC and infant. **(c)** Venn-diagram showing the number of significantly differentially expressed genes (sDEGs) for each comparison among SCs per time point and regulation type (up/down-regulated). sDEGs are determined as those with a log2-fold-change>0.25 and adjusted p<0.05, calculated using two-sided Wilcoxon rank-sum tests. **(d)** Significant overlaps (determined by Fisher's exact test, p<0.05) between sDEGs and modules for each time point and regulation type within each SC. The odds ratio (color-bar legend) represents the strength of association, and the Jaccard index (legend circles) measures the similarity between two lists.

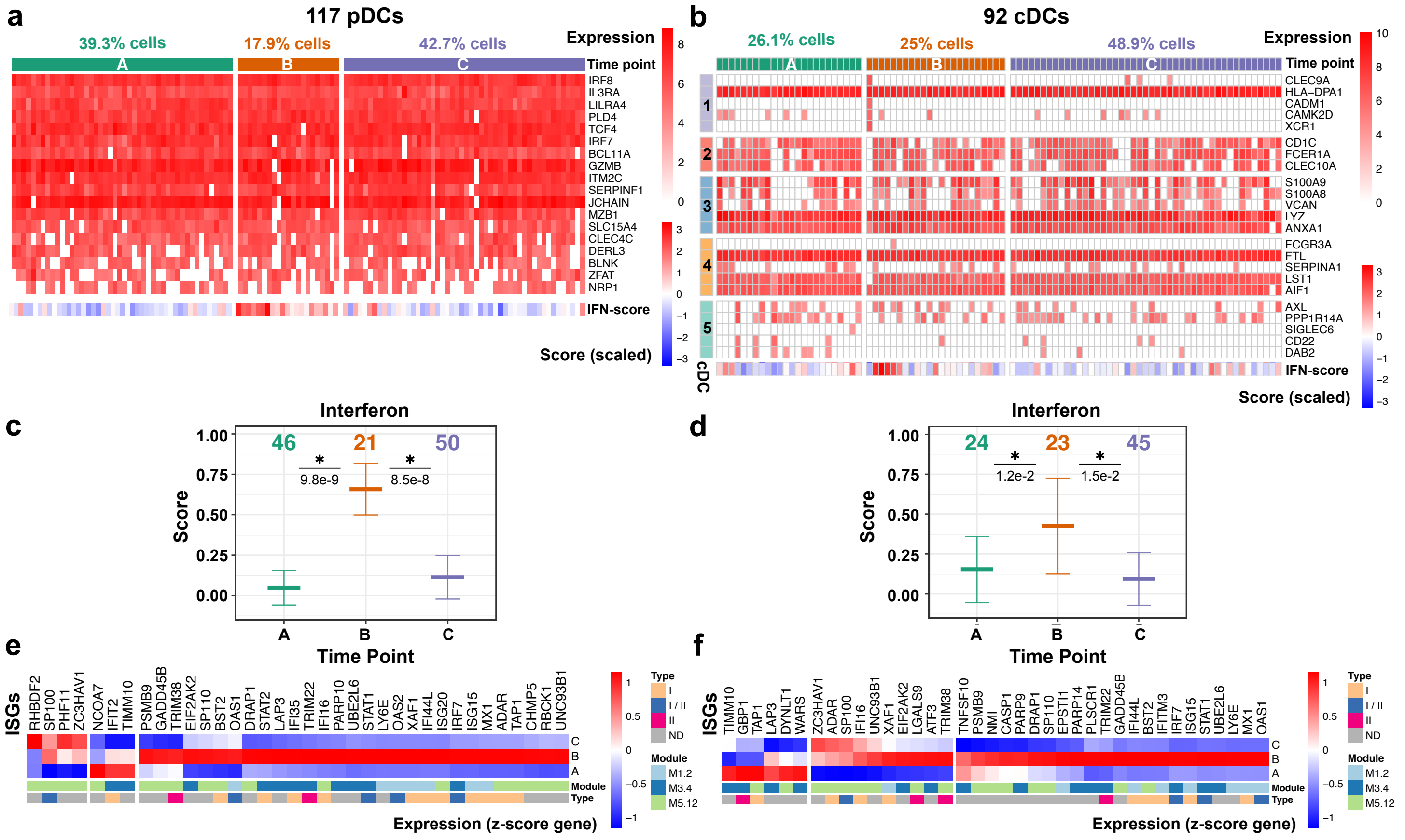

Supplementary Figure 10: Plasmacytoid dendritic cells (pDCs) and conventional dendritic cells (cDCs) panels. (a-b) Expression level of selected genes for each pDC **(a)** and cDC **(b)**. Scaled interferon scores calculated for each cell are shown in the bottom row. Proportion of cells per each time point is shown by color-coded label on top. **(c-d)** Interferon score calculated for each cell per time point for pDCs **(c)** and cDCs **(d)**. Error bar indicates standard deviation. Horizontal bar indicates mean. An asterisk (*) indicates a significant difference between two consecutive time points, determined using two-sided t-tests, with unadjusted p<0.05 (labels). **(e-f)** Average expression levels of top 30 Interferon-stimulated genes (ISGs) per each time point for pDCs **(e)** and cDCs **(f)**. The color keys on the bottom represent genes association.

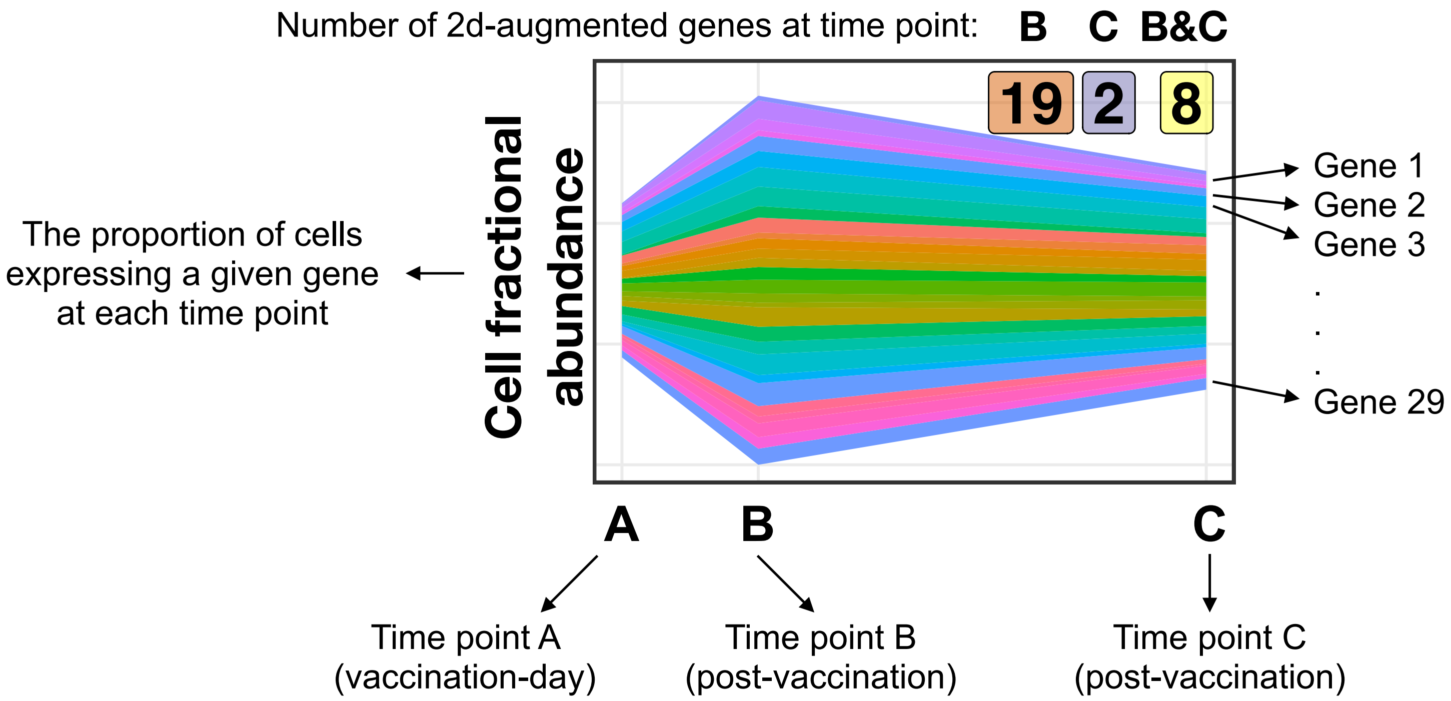

Supplementary Figure 11: Stream plot for the visualization of 2D-augmented genes. Each ribbon spans three time-points A, B, and C (x-axis) and represents an augmented gene. The thickness of each ribbon (y-axis) indicates the proportion of cells expressing the given gene at each time point. Labels at the top-right indicate the number of identified augmented genes at time points B (orange), C (purple), or both B and C (yellow).

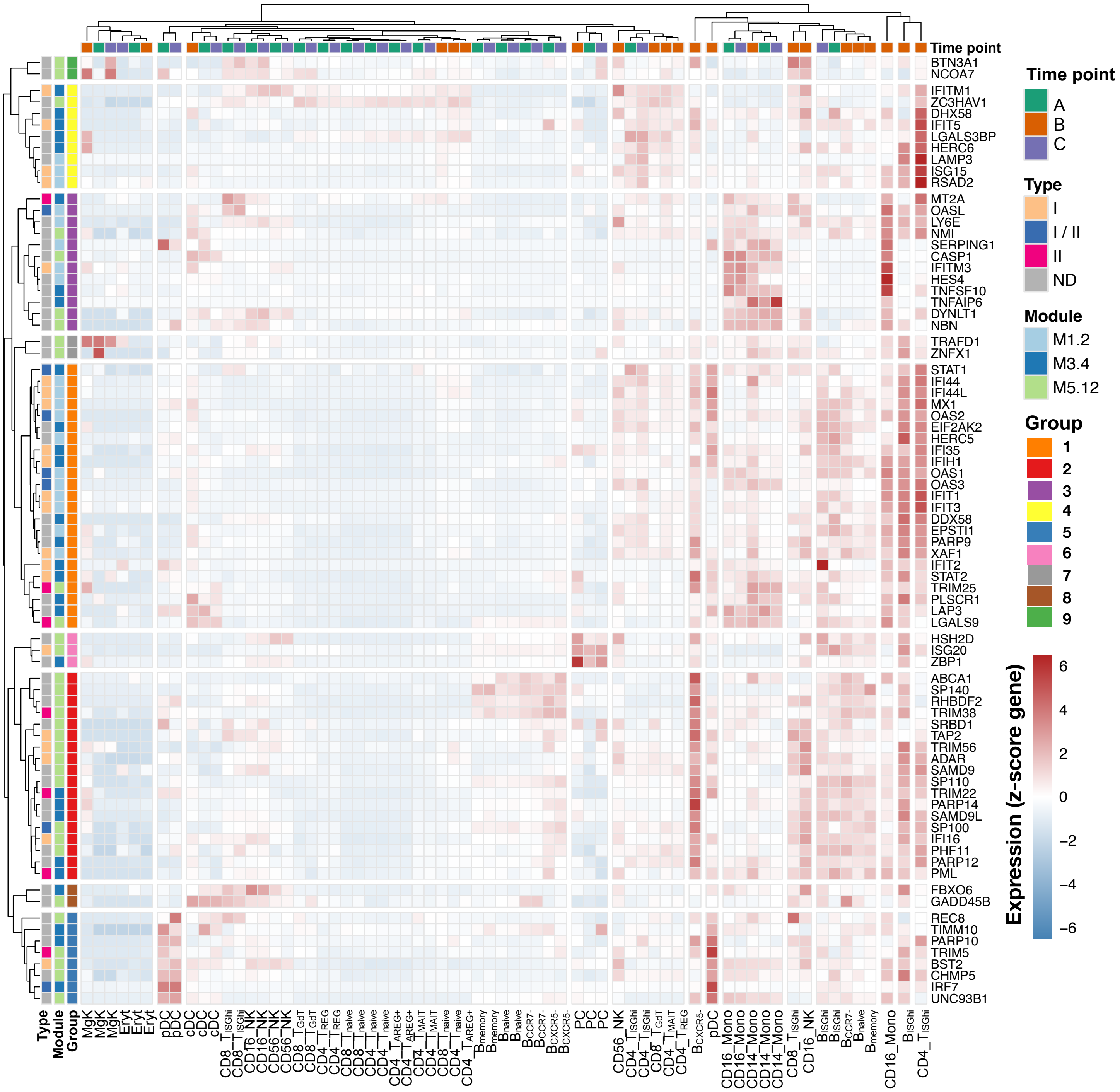

Supplementary Figure 12: Gene 2-dimension augmentation supplementary panel. Average expression levels of 2D-augmented interferon-related genes (rows) are depicted for each subcluster and time point (columns). Hierarchical clustering generates row and column dendrograms. The color in the heatmap indicates the mean expression for each entry. The left-side color keys represent gene associations, while the top color key denotes sampling time points.

References

Hao, Yuhan and Hao, Stephanie and Andersen-Nissen, Erica and Mauck III, William M and Zheng, Shiwei and Butler, Andrew and Lee, Maddie J and Wilk, Aaron J and Darby, Charlotte and Zager, Michael and others. 2021. "Integrated analysis of multimodal single-cell data." *Cell* (Elsevier) 184 (13): 3573--3587.

Stuart, Tim and Butler, Andrew and Hoffman, Paul and Hafemeister, Christoph and Papalexi, Efthymia and Mauck III, William M and Hao, Yuhan and Stoeckius, Marlon and Smibert, Peter and Satija, Rahul. 2019. "Comprehensive integration of single-cell data." *Cell* (Elsevier) 177 (7): 1888--1902.
