# Supplementary material for: Young infants display heterogeneous serological responses and extensive but reversible transcriptional changes following initial immunizations
Source: Nat Commun. 2023 Dec 2;14:7976. doi: 10.1038/s41467-023-43758-2 (PMC10693608; doi:10.1038/s41467-023-43758-2)
Supplement: Supplementary file 3 — Description of additional supplementary files [file 41467_2023_43758_MOESM3_ESM.docx]

**Description of additional supplementary files**

**Supplementary Data 1**: Bulk and single cell transcriptome cohort metadata, demographics, and associated serologic responses.

**Supplementary Data 2**: List of differential expressed gene calculated from bulk data comparing post-vaccination gene expression to the baseline.

**Supplementary Data 3**: List of differential expressed gene calculated from single cell data comparing post-vaccination gene expression to the baseline.
