# Supplementary material for: Young infants display heterogeneous serological responses and extensive but reversible transcriptional changes following initial immunizations
Source: Nat Commun. 2023 Dec 2;14:7976. doi: 10.1038/s41467-023-43758-2 (PMC10693608; doi:10.1038/s41467-023-43758-2)
Supplement: Supplementary file 7 — Reporting Summary [file 41467_2023_43758_MOESM7_ESM.pdf]

nature portfolio

Corresponding author(s): Octavio Ramilo
Last updated by author(s): Oct 28, 2023

Reporting Summary

Nature Portfolio wishes to improve the reproducibility of the work that we publish. This form provides structure for consistency and transparency in reporting. For further information on Nature Portfolio policies, see our Editorial Policies and the Editorial Policy Checklist.

Statistics

For all statistical analyses, confirm that the following items are present in the figure legend, table legend, main text, or Methods section.

n/a	Confirmed	
☐	☒	The exact sample size (*n*) for each experimental group/condition, given as a discrete number and unit of measurement
☐	☒	A statement on whether measurements were taken from distinct samples or whether the same sample was measured repeatedly
☐	☒	The statistical test(s) used AND whether they are one- or two-sided<br>*Only common tests should be described solely by name; describe more complex techniques in the Methods section.*
☐	☒	A description of all covariates tested
☐	☒	A description of any assumptions or corrections, such as tests of normality and adjustment for multiple comparisons
☒	☐	A full description of the statistical parameters including central tendency (e.g. means) or other basic estimates (e.g. regression coefficient) AND variation (e.g. standard deviation) or associated estimates of uncertainty (e.g. confidence intervals)
☐	☒	For null hypothesis testing, the test statistic (e.g. *F*, *t*, *r*) with confidence intervals, effect sizes, degrees of freedom and *P* value noted<br>*Give P values as exact values whenever suitable.*
☒	☐	For Bayesian analysis, information on the choice of priors and Markov chain Monte Carlo settings
☐	☒	For hierarchical and complex designs, identification of the appropriate level for tests and full reporting of outcomes
☒	☐	Estimates of effect sizes (e.g. Cohen's *d*, Pearson's *r*), indicating how they were calculated

Our web collection on statistics for biologists contains articles on many of the points above.

Software and code

Policy information about availability of computer code

Data collection	Clinical data was collected using the RECAP database. Laboratory data in excel dataset
Data analysis	"For single cell analysis the Illumina basecall files (BCL) were converted to fastqs using cell ranger v3.0.2 which uses bcl2fastq v2.17.1.14. FASTQ files were then alligned to the hg19 genome and transcriptome using the cellranger v3.0.2 pipeline. Scrublet phyton package version 0.2.2 was used to remove doublets. Seurat Package 4.0.3 was used for downstream gene expression analyses. Harmony R package version 1.0 used for harmonization. GeneOverlap R package was used for enrichment analysis. R version 4.2 was used for analysis. All software is open source. For bulk transcriptome analysis the Illumina GenomeStudio software was used for pre-processing the data (background subtraction and normalizations). Downstream analysis was performed in R environment. Differentially expressed genes between the groups were identified using limma package with adjusted p value less than 0.05 and fold change greater than 1.5. To assess the immune function of the differentially expressed genes between the groups we used a modular transcriptional repertoire framework (Chaussabel D. a., 2008; Chaussabel D. a., 2014)"

For manuscripts utilizing custom algorithms or software that are central to the research but not yet described in published literature, software must be made available to editors and reviewers. We strongly encourage code deposition in a community repository (e.g. GitHub). See the Nature Portfolio guidelines for submitting code & software for further information.

Data

Policy information about availability of data

All manuscripts must include a data availability statement. This statement should provide the following information, where applicable:

- Accession codes, unique identifiers, or web links for publicly available datasets
- A description of any restrictions on data availability
- For clinical datasets or third party data, please ensure that the statement adheres to our policy

Individual and aggregated raw counts are available in GEO: GSE204716. FASTQ files will be available on dbGAP (phs002926.vl.pl) upon paper acceptance. Code used for the analysis is available on GitHub: https://github.com/nourin-nn/infants_vaccines_cocktail.The bulk transcriptome data are deposited in the National Center for Biotechnology Information Gene Expression Omnibus (GSE237318). After manuscript acceptance we have instructed the databases to release all data information.

Research involving human participants, their data, or biological material

Policy information about studies with human participants or human data. See also policy information about sex, gender (identity/presentation), and sexual orientation and race, ethnicity and racism.

Reporting on sex and gender	Study participants were healthy asymptomatic 2-month old infants both males and females
Reporting on race, ethnicity, or other socially relevant groupings	These were healthy infants of all races and ethnicities recruited at the time of routine visits including vaccinations
Population characteristics	Healthy asymptomatic 2 month old infants
Recruitment	Study subjesct were recruited in the outpatient clinics at Nationwide Children's Hospital, Columbus, Ohio
Ethics oversight	The IRB at Nationwide Children's Hospital reviewed and approved the study protocol

Note that full information on the approval of the study protocol must also be provided in the manuscript.

Field-specific reporting

Please select the one below that is the best fit for your research. If you are not sure, read the appropriate sections before making your selection.

☒ Life sciences ☐ Behavioural & social sciences ☐ Ecological, evolutionary & environmental sciences

For a reference copy of the document with all sections, see nature.com/documents/nr-reporting-summary-flat.pdf

Life sciences study design

All studies must disclose on these points even when the disclosure is negative.

Sample size	This was an observational clinical study including 30 infants who were fo1lowed longitudinally. we obtained 3 sample from each participant.
Data exclusions	No data were excluded. In a few occasions because of the limited blood volume available were were not able to to perform all assays in each of the samples.
Replication	The bulk and single cell transcriptome from each individual infant samples were analyzed individually and asa group to validate the consistency of the findings.
Randomization	As this was an observational study there was no randomization with respect to patient enrollment."
Blinding	"The analytical team was independent and separate form the clinical team and had limited demographic data when analyzing the different immunologic assays, as they had to order the samples for analysis

Reporting for specific materials, systems and methods

We require information from authors about some types of materials, experimental systems and methods used in many studies. Here, indicate whether each material, system or method listed is relevant to your study. If you are not sure if a list item applies to your research, read the appropriate section before selecting a response.

Materials & experimental systems

n/a	Involved in the study
☒	☐ Antibodies
☒	☐ Eukaryotic cell lines
☒	☐ Palaeontology and archaeology
☒	☐ Animals and other organisms
☐	☒ Clinical data
☒	☐ Dual use research of concern
☒	☐ Plants

Methods

n/a	Involved in the study
☒	☐ ChIP-seq
☒	☐ Flow cytometry
☒	☐ MRI-based neuroimaging

Clinical data

Policy information about clinical studies

All manuscripts should comply with the ICMJEguidelines for publication of clinical research and a completedCONSORT checklist must be included with all submissions.

Clinical trial registration	Please note this was an observational study and participating infants received all the standard of care vaccines as recommended at 2 months of age. Since according to NIH guidelines this was not a clinical trial, we thouhgt the registration on clnicla trials.gov was not required.
Study protocol	The study protocol was reviewed and approved by the NCH IRB. A summary of the protocol is included in the manuscript.
Data collection	Data were collected upon enrollment and after completion of the follow up visits at NCH Clinical Research Department.
Outcomes	Outcomes of the study included the vaccine-induced antibody responses and the transcriptional changes.

Plants

Seed stocks	*Report on the source of all seed stocks or other plant material used. If applicable, state the seed stock centre and catalogue number. If plant specimens were collected from the field, describe the collection location, date and sampling procedures.*
Novel plant genotypes	*Describe the methods by which all novel plant genotypes were produced. This includes those generated by transgenic approaches, gene editing, chemical/radiation-based mutagenesis and hybridization. For transgenic lines, describe the transformation method, the number of independent lines analyzed and the generation upon which experiments were performed. For gene-edited lines, describe the editor used, the endogenous sequence targeted for editing, the targeting guide RNA sequence (if applicable) and how the editor was applied.*
Authentication	*Describe any authentication procedures for each seed stock used or novel genotype generated. Describe any experiments used to assess the effect of a mutation and, where applicable, how potential secondary effects (e.g. second site T-DNA insertions, mosiacism, off-target gene editing) were examined.*
